# Antimicrobial Susceptibility of *Bordetella bronchiseptica* Isolates from Swine and Companion Animals and Detection of Resistance Genes

**DOI:** 10.1371/journal.pone.0135703

**Published:** 2015-08-14

**Authors:** Sandra Prüller, Ulrike Rensch, Diana Meemken, Heike Kaspar, Peter A. Kopp, Günter Klein, Corinna Kehrenberg

**Affiliations:** 1 Institute for Food Quality and Food Safety, University of Veterinary Medicine Hannover, Foundation, Bischofsholer Damm 15, 30173 Hannover, Germany; 2 Federal Office of Consumer Protection and Food Safety (BVL), Berlin, Germany; 3 Vet Med Labor GmbH, Division of IDEXX Laboratories, Ludwigsburg, Germany; Institut National de la Recherche Agronomique, FRANCE

## Abstract

*Bordetella bronchiseptica* causes infections of the respiratory tract in swine and other mammals and is a precursor for secondary infections with *Pasteurella multocida*. Treatment of *B*. *bronchiseptica* infections is conducted primarily with antimicrobial agents. Therefore it is essential to get an overview of the susceptibility status of these bacteria. The aim of this study was to comparatively analyse broth microdilution susceptibility testing according to CLSI recommendations with an incubation time of 16 to 20 hours and a longer incubation time of 24 hours, as recently proposed to obtain more homogenous MICs. Susceptibility testing against a panel of 22 antimicrobial agents and two fixed combinations was performed with 107 porcine isolates from different farms and regions in Germany and 43 isolates obtained from companion animals in Germany and other European countries. Isolates with increased MICs were investigated by PCR assays for the presence of resistance genes. For ampicillin, all 107 porcine isolates were classified as resistant, whereas only a single isolate was resistant to florfenicol. All isolates obtained from companion animals showed elevated MICs for β-lactam antibiotics and demonstrated an overall low susceptibility to cephalosporines. Extension of the incubation time resulted in 1–2 dilution steps higher MIC_50_ values of porcine isolates for seven antimicrobial agents tested, while isolates from companion animals exhibited twofold higher MIC_50/90_ values only for tetracycline and cefotaxime. For three antimicrobial agents, lower MIC_50_ and MIC_90_ values were detected for both, porcine and companion animal isolates. Among the 150 isolates tested, the resistance genes *bla*_BOR-1_ (n = 147), *bla*_OXA-2_, (n = 4), *strA* and *strB* (n = 17), *sul1* (n = 10), *sul2* (n = 73), *dfrA7* (n = 3) and *tet*(A) (n = 8) were detected and a plasmid localisation was identified for several of the resistance genes.

## Introduction

The first report of *B*. *bronchiseptica* as causative agent of a respiratory tract infection in a dog was published in 1911 [1]. Since then, it has been shown that various mammals, such as dogs, cats, rabbits, and horses can be infected with this bacterial pathogen [2–7]. Infections with *B*. *bronchiseptica* in these mammals can vary from asymptomatic cases to severe bronchopneumonia [8]. Moreover, *B*. *bronchiseptica* is currently a well-known pathogen in swine and is associated with a disease designated as atrophic rhinitis [9]. In the pathogenesis of atrophic rhinitis, infection with *B*. *bronchiseptica* predisposes the animals to infections with toxigenic strains of *Pasteurella multocida*. This may lead to a severe form of the disease [10–11]. Infections in immunocompromised human patients, typically manifested as bronchitis, pneumonia, or whooping cough, have also been reported. Some of these reports implicated an association of human disease with the exposure to infected companion animals [12–14]. However, there have been some cases of nosocomial transmission of *B*. *bronchiseptica* between humans [14–15].

Bacterial respiratory tract infections of animals are usually treated with antimicrobial agents, such as tetracyclines, fluoroquinolones, macrolides, penicillin, phenicols or a combination of trimethoprim and sulphonamides [16–18]. Nevertheless, only few studies are available on the antimicrobial susceptibility status of *B*. *bronchiseptica* isolates from swine or companion animals in Europe or other countries [7, 18–22]. A comparison between results from these studies can not be reliably performed due to the different methods of susceptibility testing used [22–25]. However, decreased antimicrobial susceptibility of *B*. *bronchiseptica* isolates has been reported for some β-lactam antibiotics such as penicillins and cephalosporines (e.g. ceftiofur and cefalothin) due to production of β-lactamases or an overall low membrane permeability to cephalosporines [18, 26–28]. In addition, *B*. *bronchiseptica* isolates with elevated MIC values of trimethoprim/sulfamethoxazole and tetracycline have also been demonstrated [18].

So far, there is a CLSI-approved performance standard of broth microdilution susceptibility testing for non-fastidious, rapidly replicating bacteria given in document VET01-A4 [29]. It has recently been shown by using a limited number of isolates, that media, inoculum density and incubation temperature are generally suitable for *B*. *bronchiseptica* isolates. However, it could be demonstrated in the previous study that reading the results after 24 hours incubation lead to more homogenous MIC values compared to the standard 16 to 20 hours incubation time and the applicability of the slightly modified method was confirmed in an interlaboratory comparison trial [30]. By investigating a larger number of field isolates, it should be clarified if reading the results after 24 hours would lead to a difference in the classification of *B*. *bronchiseptica* isolates as susceptible or resistant and it should be investigated if the extended incubation time results in deviating MIC values. In addition, it is important to get an overview of the susceptibility of more recently collected *B*. *bronchiseptica* isolates from swine and companion animals and to detect trends in resistance development.

Therefore, the aims of this study were (i) to determine MIC values of more recently collected *B*. *bronchiseptica* isolates from swine and companion animals from Germany and different European countries against a broad panel of antimicrobial agents and (ii) to comparatively analyse MIC values after 16 to 20 and 24 hours of incubation. Furthermore, isolates with elevated MIC values were investigated for the presence of resistance genes.

## Materials and Methods

### Bacterial isolates

A total of 150 *B*. *bronchiseptica* field isolates were included in the study. The isolates were kindly provided from diagnostic laboratories in Germany and were collected between 2010 and 2012 from diseased and healthy animals. All isolates were collected on the basis of one isolate per herd and per year. The study included 107 isolates from swine (85 isolates from diseased animals, 2 isolates from healthy animals and 20 isolates from animals without preliminary report regarding the health status) collected in distinct geographical regions with a high density of swine in Germany and 43 isolates from companion animals collected in different European countries (Germany, Denmark, Sweden, France and Italy). Companion animal isolates were collected from horses (n = 24), dogs (n = 8), rabbits (n = 8), cats (n = 2) and a ferret (n = 1). Species identification of all isolates was confirmed by a *B*. *bronchiseptica*-specific PCR assay amplifying the upstream region of the flagellin gene *fla*A (237bp) as described earlier [31]. The isolates were cultured on Columbia Blood Agar (Oxoid Germany GmbH, Wesel, Germany) for 24 hours ± 2 hours in ambient air at 35°C ± 1.0°C.

### Susceptibility Testing

Minimum inhibitory concentrations (MICs) were determined by broth microdilution susceptibility testing in accordance with the Clinical and Laboratory Standards Institute standard VET01-A4 for rapidly growing aerobic bacteria isolated from animals [29]. Customised microtiter plates were used to assess MICs against a panel of 24 antimicrobial agents and antibiotic combinations (Sensititre, Trek Diagnostic Systems, East Grinstead, UK). It should be noted that this panel comprised some antimicrobial agents which are currently not licensed for animals (imipenem, ciprofloxacin, nalidixic acid, cefotaxime) or food producing animals (chloramphenicol). Microtiter plates were incubated at 35°C ± 1°C and the results were read visually by the same person after 18 hours ± 2 hours and, for comparison reasons, after 24 hours of incubation [29]. In the CLSI-Document VET01-A4, an incubation time of 16 to 20 hours is recommended for non-fastidious bacteria. However, as it has recently been shown, an incubation time of 24 hours is advantageous for a standardised susceptibility testing of *B*. *bronchiseptica* isolates and results in significantly more homogeneous MIC values [30]. Hence, the extended incubation time was also included and compared to results obtained after 20 hours, which is at the upper end of the CLSI recommended range. For quality control purposes, *Escherichia coli* strain ATCC 25922 was used and *B*. *bronchiseptica* CFU/ml were determined in every test run to confirm the final inoculum concentration of approximately 5 x 10^5^ CFU/ml.

### Comparative statistical analysis

A comparison of MIC values obtained after 20 hours and 24 hours of incubation was performed by using the nonparametric Wilcoxon matched pair test, with differences considered significant at *P≤0*.*05*.

### PCR amplification of antimicrobial resistance genes

Bacterial DNA template was prepared by using the DNeasy blood and tissue kit following the manufacturer´s instructions (Qiagen, Hilden, Germany). PCR amplification for the presence of resistance genes included all isolates classified as resistant to an antimicrobial agent as well as isolates with elevated MICs to antimicrobials (isolates exhibiting MICs in the right group of a bimodal distribution), for which no approved breakpoint is available. The PCR primers and amplicon sizes are shown in Table 1. The PCR primers were either chosen from published PCR assays or were selected from sequences available in the Genbank database. PCR screening included detection of the β-lactam resistance-mediating genes *ampC*, *bla*_BOR-1_, *bla*_CMY_, *bla*_OXA_, *bla*_*TEM*,_ *bla*_CTX_, *bla*_PSE_ and *bla*_SHV_ [32–34], the phenicol resistance genes *cfr*, *cmlA*, *cmlB1*, *catA1*, *catA2*, *catA3*, *catB2*, *catB*3, *fexA* and *floR* [26, 35–37], the aminoglycoside resistance-mediating genes *aph*(3´)-Id, *aph*-3-I, *aphA*-3 and *aph*-A6, *strA* and *strB* [38–39], the tetracycline resistance genes *tet*(A), *tet*(B), *tet*(C), *tet*(D), *tet*(E), *tet*(G), *tet*(H), *tet*(L), *tet*(M) and *tet*(O) [40–43] and PCR assays for the macrolide resistance genes *erm*(42), *mph*(E) and *msr*(E), which are associated with elevated MICs for tilmicosin [44]. In addition, PCR assays were performed to detect the sulphonamide resistance genes *sul1*, *sul2* and *sul3* and the trimethoprim resistance genes *dfrA1*, *dfrA5/A14*, *dfrA7/A17* and *dfrB1* [35, 45–46]. The PCR protocol for the detection of resistance genes included the use of Taq DNA Polymerase (Fisher Scientific GmbH, Schwerte, Germany) in a total volume of 25 μl reaction mixture. As the *dfrA7/A17* PCR assay detects both resistance genes, sequencing of the PCR amplicons was conducted and nucleotide sequences were analyzed by using the program nucleotide BLAST (http://blast.ncbi.nlm.nih.gov/Blast.cgi).

**Table 1:** PCR primers designed for this study.

Gene	Primers	Fragment size (bp)	Reference
*aph(3`)-Id*	fw: 5´-cgg cag caa tgt tta tcg -3'	600	this study
	rv: 5´-aga cgg ttc cag agt atg gc-3'		
*aph-3-I*	fw: 5'-caa tca ggt gcg aca atc tat c-3'	637	this study
	rv: 5'-gcc gtt tct gta atg aag gag -3'		
*aphA-3*	fw: 5´-acc tat gat gtg gaa cgg g-3'	513	this study
	rv: 5´-gca gaa ggc aat gtc ata cc-3'		
*aphA-6*	fw: 5'-cat aca gtg tct ctc gtg aag c-3'	534	this study
	rv: 5'-cat cct ctc tta ggc aac g-3'		
*bla*_BOR-1_	fw: 5'—acg aac gct ttc cga tgt g—3'	650	this study
	rv: 5'—ttc tgc cag cac agc att c—3'		
*catB2*	fw: 5'—tga gca ggt gaa gaa tcc g—3'	565	this study
	rv: 5'—acg ata cag gct ggc aat g—3'		
*catB3*	fw: 5'—tct gag caa gtg aag aac cc—3'	461	this study
	rv: 5'—tca tcg gtg aag cgt ttc—3'		
*cmlB1*	fw: 5'—cga ctt gtt ggc atc act c—3'	1081	this study
	rv: 5'—tgc ggc ata cag gca cag ac—3'		

### Detection of plasmid-located antimicrobial resistance genes

Isolates tested positive by PCR for one of the aforementioned resistance genes were investigated by Southern blotting to determine a possible localisation of the genes on plasmids. For this, plasmid DNA was extracted by using a combination of the alkaline lysis method and a phenol-chloroform-extraction as previously described [47]. Subsequently, plasmids were separated in a 1% agarose gel and transferred to a nylon membrane (Roche Diagnostics Deutschland GmbH, Mannheim, Germany). Hybridization was performed with gene probes consisting of PCR-amplified internal fragments of the antimicrobial resistance genes *bla*_OXA_, *sul1*, *sul2*, *strA* and *strB*, *tet*(A) The PCR amplicons were non-radioactively labelled using the PCR DIG Probe Synthesis Kit (Roche Diagnostics GmbH, Mannheim, Germany).

## Results

### MIC distribution and classification of *B*. *bronchiseptica* isolates

**Fig 1:**
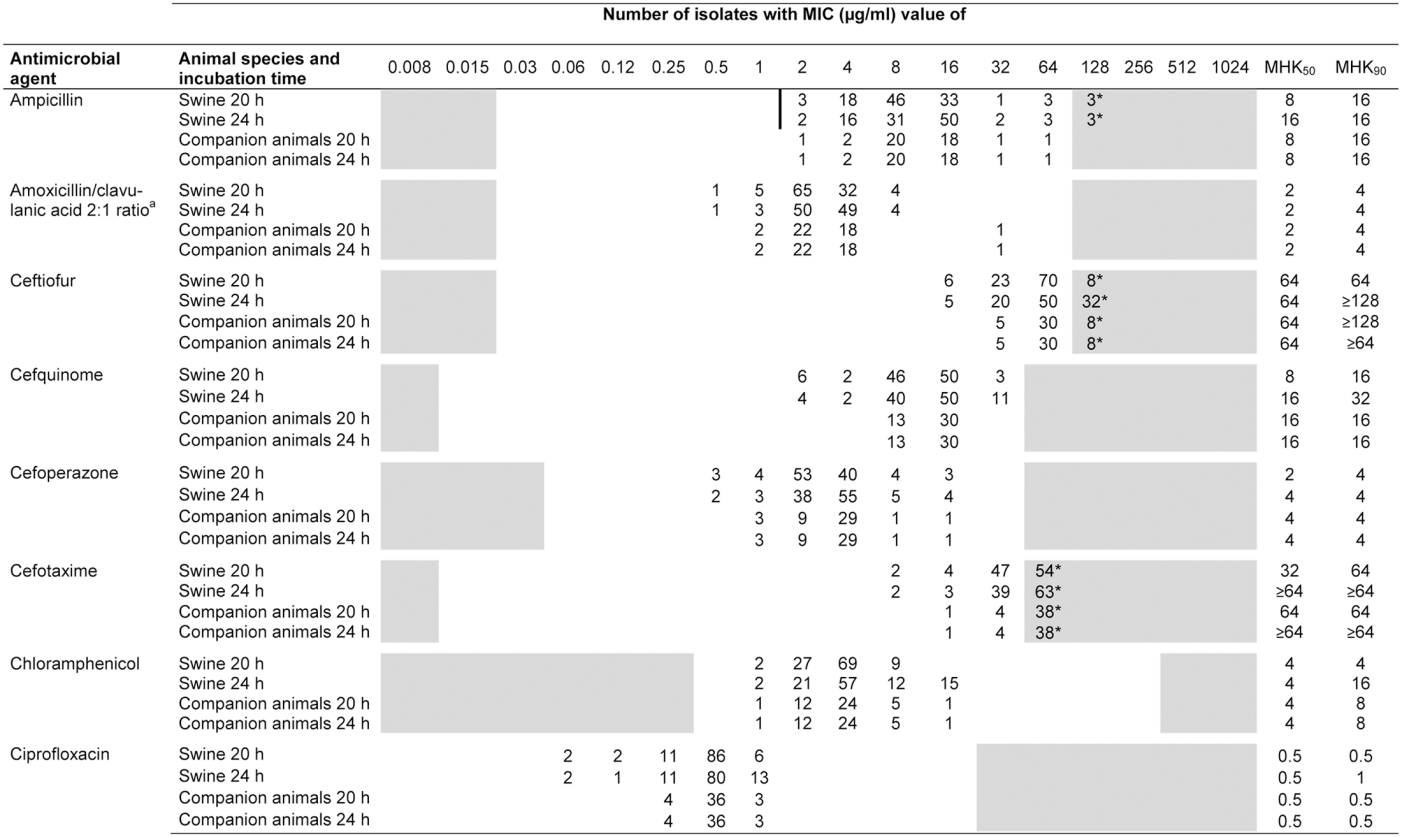
MIC distribution of 150 *B*. *bronchiseptica* isolates from swine and companion animals after 20 and 24 hours of incubation. ^a^Data represent the concentrations of amoxicillin; *Asterisked numbers indicate the number of isolates exhibiting MIC values equal to or higher /lower than concentrations of the test range. The white areas represent the tested range of an antimicrobial agent and bars indicate the CLSI recommended breakpoints for resistance of an antimicrobial agent.

**Fig 2:**
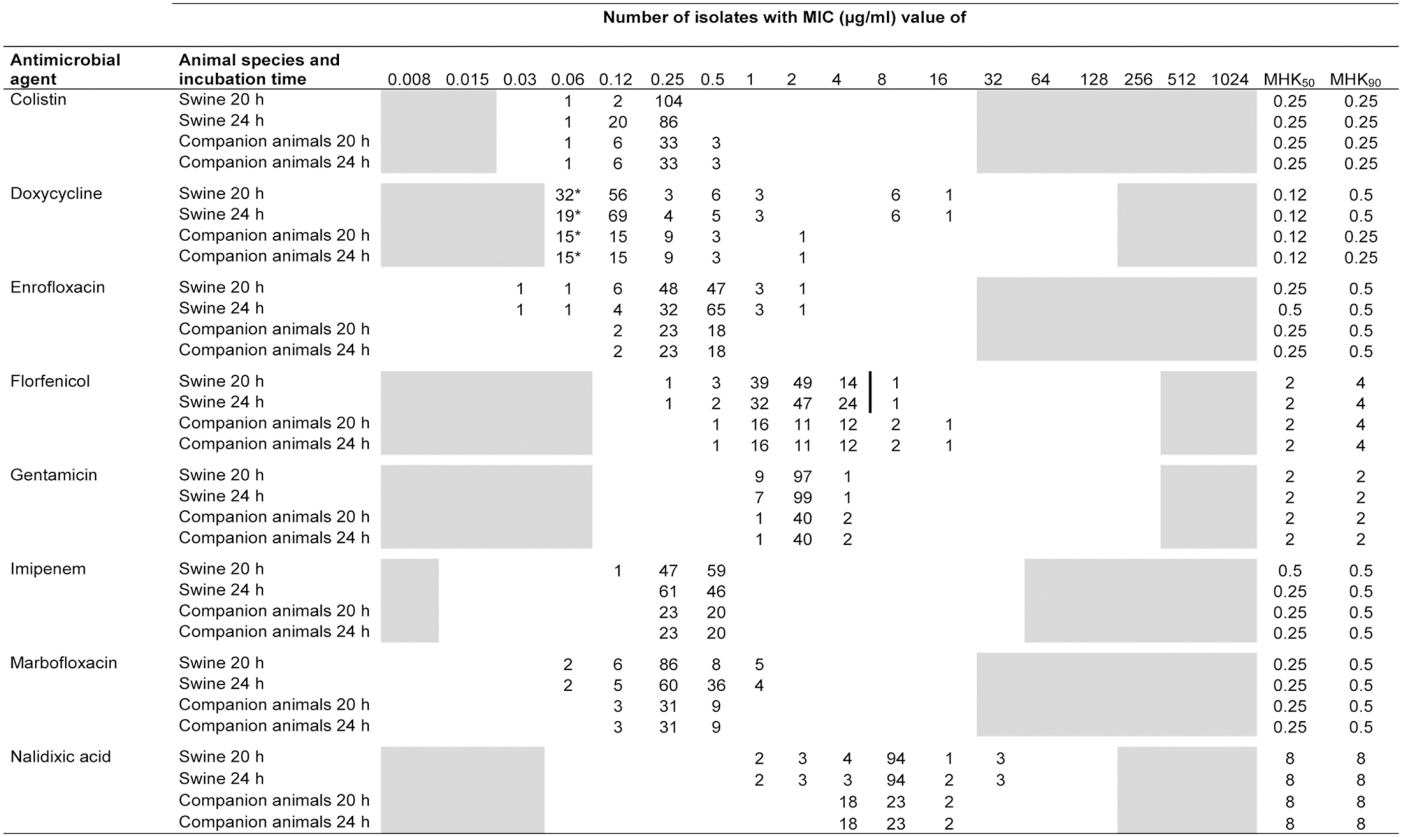
MIC distribution of 150 *B*. *bronchiseptica* isolates from swine and companion animals after 20 and 24 hours of incubation. *Asterisked numbers indicate the number of isolates exhibiting MIC values equal to or higher /lower than concentrations of the test range. The white areas represent the tested range of an antimicrobial agent and bars indicate the CLSI recommended breakpoints for resistance of an antimicrobial agent.

**Fig 3:**
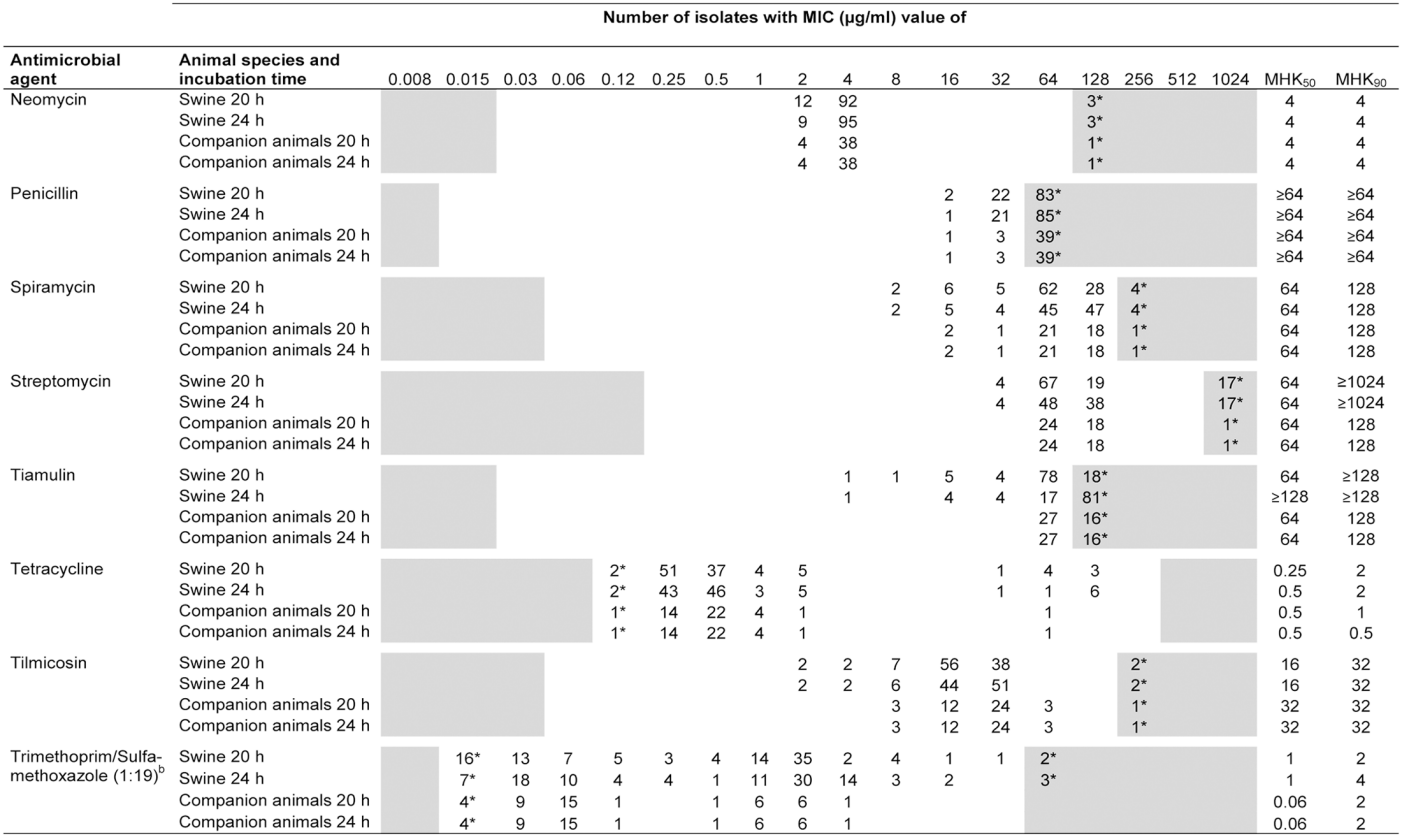
MIC distribution of 150 *B*. *bronchiseptica* isolates from swine and companion animals after 20 and 24 hours of incubation. ^b^Data represent the concentrations of trimethoprim; *Asterisked numbers indicate the number of isolates exhibiting MIC values equal to or higher /lower than concentrations of the test range. The white areas represent the tested range of an antimicrobial agent.

The MIC values of the 107 porcine and 43 companion animal *B*. *bronchiseptica* isolates are summarized in Figs 1–3. A unimodal distribution of MIC values was detected for some antimicrobial agents tested, such as chloramphenicol, gentamicin and marbofloxacin, while for other antibiotics such as neomycin, tetracycline or tilmicosin, a clear bimodal distribution of MICs was observed. A classification of the isolates as susceptible, intermediate or resistant has proved to be difficult, since CLSI approved *B*. *bronchiseptica*-specific breakpoints are currently available only for ampicillin, florfenicol and tulathromycin and the indication of respiratory tract infections in swine [29]. Due to a fixed microtiter plate layout, MIC values were obtained for ampicillin and florfenicol, while MICs of tulathromycin were not included.

In terms of the CLSI recommended 16 to 20 hours incubation time for rapidly growing bacteria, all 107 porcine isolates of our strain collection were classified as ampicillin resistant. In contrast, only a single porcine isolate (0.9%) was categorized as florfenicol resistant and 13.1% (14 isolates) were intermediate resistant to florfenicol. The remaining 43 isolates originating from companion animals exhibited MIC values of ampicillin and florfenicol in a similar range than porcine isolates (2 to 64 μg/ml ampicillin and 0.5 to 16 μg/ml florfenicol, respectively) and, hence, are presumably in the category of ampicillin (all 43 isolates) and florfenicol (three isolates) resistance as well. All isolates with elevated MICs of florfenicol (≥8 μg/ml) also exhibited elevated MICs of chloramphenicol (8 and 16 μg/ml, respectively).

With regard to the β-lactam antibiotics, a difference between ampicillin and amoxicillin/clavulanic acid MIC values was detected with MIC_50_ and MIC_90_ values of ampicillin being fourfold higher compared to a combination of amoxicillin with a β-lactamase inhibitor. In addition, overall high MIC values of penicillin and cephalosporins (e.g. ceftiofur, cefquinome, cefotaxime) were recorded for porcine and companion animal isolates. For tetracycline and doxycycline, a clear bimodal distribution of MICs was detected with overall nine and seven isolates showing MICs of ≥32 μg/ml tetracycline and ≥8 μg/ml doxycycline, respectively. A bimodal distribution was also detected for the aminoglycoside neomycin with the majority of MICs ranging between 2 to 4 μg/ml and only few isolates exhibited higher MICs of ≥128 μg/ml. A similar situation was seen for streptomycin with 132 isolates (88% of isolates) exhibiting an MIC value of ≤128 μg/ml and 18 isolates of ≥1024 μg/ml. For the third aminoglycoside tested, gentamicin, a more uniform distribution of MICs was recorded (range 1 to 4 μg/ml). For both fluoroquinolones, enrofloxacin and marbofloxacin, low MIC_50_ and MIC_90_ values of 0.25 and 0.5 μg/ml were observed, while for tilmicosin, 147 isolates showed MIC values between 2 and 64 μg/ml and three isolates MICs of ≥256 μg/ml. In this strain collection, the broadest distribution of MICs was detected for the antibiotic combination trimethoprim/sulfamethoxazole (1:19), with isolates exhibiting MICs between 0.015/0.3 μg/ml and ≥64/1216 μg/ml.

### Comparative analysis of MIC values

A comparison of results from susceptibility testing after 20 hours (upper limit of the CLSI recommended incubation times for non-fastidious bacteria) and 24 hours incubation time as previously proposed for *B*. *bronchiseptica* was performed [30]. Out of 24 antimicrobial agents tested, the MIC_50_ values of porcine isolates showed slightly (1 to 2 dilution steps) higher values after 24 hours incubation for ampicillin, cefquinome, cefoperazone, cefotaxime, enrofloxacin, tiamulin, and tetracycline, while MIC_90_ values of ceftiofur, cefquinome, cefotaxime, chloramphenicol, ciprofloxacin, and trimethoprim/sulfamethoxazole increased by one dilution step after 24 hours of incubation. In contrast, isolates from companion animals showed twofold higher MIC_50_ and MIC_90_ values only for cefotaxime and tetracycline when using the extended incubation time. However, for ceftiofur, imipenem and tetracycline lower MIC_50_ and MIC_90_ values were detected after 24 hours incubation for porcine and companion animal isolates.

Statistical analysis of MIC values obtained from all 150 isolates for the 24 antimicrobial agents tested revealed that MICs for 18 antimicrobials were slightly, but statistically significant higher after 24 hours incubation when compared to 20 hours. For the remaining six antimicrobial agents, colistin, gentamicin, imipinem, nalidixic acid, neomycin and penicillin, there was no significant difference in MICs between 20 and 24 hours incubation.

### Detection of antimicrobial resistance genes and localisation on plasmids

**Fig 4:**
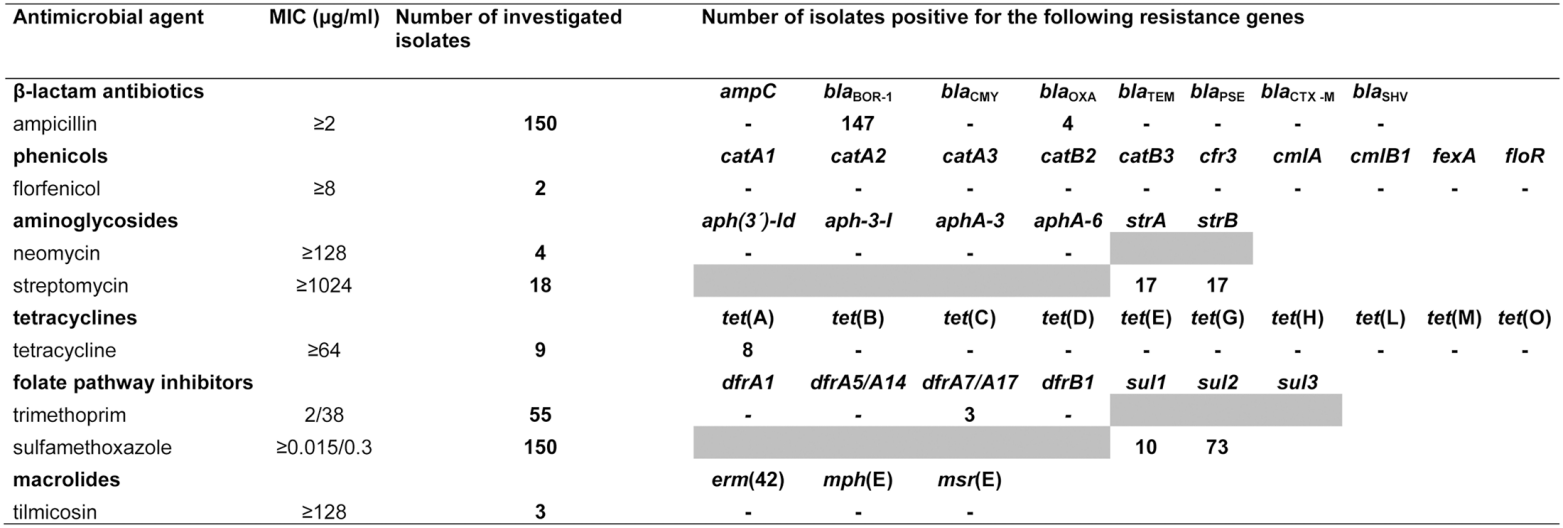
Investigated isolates and detected resistance genes.

PCR analysis of whole cell DNA including all 107 ampicillin resistant isolates from swine and 43 isolates from companion animals with elevated MICs (≥2 μg/ml ampicillin) demonstrated that 146 isolates harbour the *bla*_BOR-1_ β-lactamase gene (Fig 4). Of these, three isolates with an MIC of ≥128 μg/ml and a single isolate exhibiting a lower MIC of 16 μg/ml additionally carried the gene *bla*_OXA_. In all four isolates, the *bla*_OXA_ gene was located on plasmids with estimated sizes of approximately 50–70 kb. Sequencing of the *bla*_OXA_ PCR amplicons detected 100% nucleotide sequence identity to an internal region of the *bla*_OXA-2_ gene from *B*. *bronchiseptica* plasmid R906 (accession number KF743818.1). In contrast, no amplification product was obtained from isolates with MICs of ≥8 μg/ml florfenicol or ≥16 μg/ml chloramphenicol for any of the phenicol resistance genes tested. In 17 out of 18 isolates with an MIC of ≥1024 μg/ml streptomycin, the resistance genes *strA* and *strB* were detected, while neither *aph(3´)-Id*, *aph-3-I*, *aphA-3* nor *aphA-6* were present in isolates with elevated MICs of streptomycin (≥1024 μg/ml) or neomycin (≥128 μg/ml). Fourteen isolates were found to carry *strA* and *strB* on plasmids either varying in size between 5 to 7 kb (7 isolates) or between 50 to 90 kb (7 isolates). No *erm*(42), *mph*(E) and *msr*(E) genes could be found in any of the three isolates with an MIC of ≥128 for tilmicosin. As a broad distribution of MICs was detected for the folate pathway inhibitor combination trimethoprim/sulfamethoxazole, all isolates were included in PCR screening assays. In 83 out of 150 isolates tested, the sulphonamide or trimethoprim resistance genes *sul1*, *sul2* or *dfrA7/A17* were detected. Of these, three porcine isolates carried *sul1* in addition to *dfrA7/A17* and exhibited a comparatively high MICs of 16/304 to ≥32/608 μg/ml whereas seven isolates with MICs between ≤0.015/0.3 and 2/38 μg/ml carried only *sul1*. Nucleotide sequence analysis of *dfrA7/A17* PCR amplicons revealed 99% identities to internal regions of *dfrA7* genes, e.g. from *E*. *coli* (GenBank accession no. CP011331.1) and *Salmonella enterica* (GenBank accession no. KM823525.1). Furthermore, the *sul2* gene was present in 73 isolates exhibiting MICs of 0.03/0.6 to 8/152 μg/ml trimethoprim/sulfamethoxazole and none of the isolates additionally carried one of the trimethoprim resistance genes tested. Due to the high amount of *sul1* and *sul2* positive isolates, a selection of eight *B*. *bronchiseptica* with MICs of ≥8/152 μg/ml was chosen for plasmid investigation. A plasmid localisation was confirmed in all eight isolates with plasmids ranging in size between 50 and 90 kb (3 isolates) or between 5 to 7 kb (5 isolates). PCR products for the tetracycline resistance gene *tet*(A) were detected in eight isolates with MIC values of ≥64 μg/ml tetracycline, while a single isolate showed MICs of 32 μg/ml tetracycline and 1 μg/ml doxycycline and did not carry any of the resistance genes screened by PCR. None of the isolates was positive for tetracycline resistance genes of the classes B-E, G, H, L, M and O. All *tet*(A) positive isolates carried the gene on plasmids of approximately 50 to 90 kb.

## Discussion

A total of 150 *B*. *bronchiseptica* isolates from swine and companion animals were tested by broth microdilution susceptibility testing against a broad panel of antimicrobial agents to get an overview of the susceptibility status of current isolates.

For the majority of the 24 antimicrobial agents tested, a unimodal distribution of MIC values was detected, whereas for a very limited number of antimicrobials, a clear bimodal distribution (e.g. tetracycline, streptomycin) or a wide range of MICs (trimethoprim/sulfamethoxazole) was observed. A bimodal distribution is suggested to be indicative for the presence of isolates forming a resistant subpopulation of bacteria [48]. However, in the case of ampicillin, all porcine isolates were classified as resistant according to CLSI breakpoints despite the more or less unimodal distribution of MICs [29]. This classification of isolates based on a clinical breakpoint for resistance was supported by results from PCR assays, which detected a *bla*_BOR-1_ β-lactamase gene in all 107 isolates from swine. As *B*. *bronchiseptica* specific clinical breakpoints have been assigned only for ampicillin, florfenicol and tulathromycin for the indication of swine respiratory disease, a classification for most of the antimicrobial agents tested and of companion animal isolates was precluded [29].

Comparing the MIC_50_ and MIC_90_ values obtained in this study (after 20 hours incubation) to results from other studies focusing on porcine *B*. *bronchiseptica* isolates, it becomes apparent that current isolates from Australian swine showed identical values for ampicillin, ceftiofur and tilmicosin, while slightly higher MIC_50_ values were reported for florfeniol and tetracycline, and slightly lower MIC_50_values for penicillin [23]. Results from the Australian study and from other studies confirmed our observation of a broad distribution of *B*. *bronchiseptica* MIC values for trimethoprim/sulfamethoxazole and generally high MICs for β-lactam antibiotics [7, 18, 20–21, 23, 25–26, 28, 34, 49]. A former study from Germany investigated MICs of isolates collected about 10 years before (between 2000 and 2003) and presented MIC_50_ and MIC_90_ values similar to MICs of this study, except for ceftiofur, neomycin, tetracycline, streptomycin and trimethoprim/sulfamethoxazole for which the MIC_90_ values seem to have increased by two dilution steps over recent years [18]. This increase in MICs might indicate a development towards elevated MICs in *B*. *bronchiseptica*. Another study from 2000 investigated MICs of porcine respiratory tract pathogens and showed one dilution step higher MIC_50_ and MIC_90_ values for tilmicosin compared to the German isolates of the present study [50]. A comparison of MIC values obtained from companion animal isolates seems to be more difficult, because many previous studies were performed by using the disk diffusion method [22, 51–52]. However, two studies from 1997 and 2000 used the agardilution and E-test methods and MIC_90_ values of *B*. *bronchiseptica* isolates, which were almost entirely obtained from cats or dogs, were similar to MIC_90_ values from companion animals in this study. Greater discrepancies were seen only for amoxicillin/clavulanic acid and doxycycline with eightfold higher MIC_90_ values in the previous studies [7, 20]. Another study investigated 42 *B*. *bronchiseptica* isolates collected between 2004 and 2006 from respiratory tract infections of dogs and cats. The MICs were comparable to the values from companion animals in the present study, with the exception of trimethoprim/sulfamethoxazole, for which higher MIC_50_ and MIC_90_ values (sixfold and twofold, respectively) have previously been reported [53]. However, it should be noted that the majority of companion animal isolates of the present study were obtained from horses, and for some animal species such as cats and ferrets, just a few isolates were available. Therefore, the distribution of MIC values and comparisons to previous studies may hide differences in antimicrobial susceptibility between isolates from various animal species. Although it would be of interest, a comparison of isolates from diseased and healthy swine was not possible due to the limited number of isolates obtained from healthy animals.

For the treatment of respiratory tract infections of swine, antimicrobial agents such as tetracyclines, doxycycline, tiamulin, amoxicillin and the combination trimethoprim/sulfamethoxazole were frequently used [54, 55]. In contrast, treatment of companion animals comprises also more expensive antimicrobials (e.g. newer generation cephalosporines) or antimicrobials that are not licensed for food-producing animals (e.g. chloramphenicol) [56]. Comparative analysis of MIC_50_ and MIC_90_ values revealed slightly higher MICs for tetracycline, doxycycline, tiamulin (MIC_90_) and a fourfold higher trimethoprim/sulfamethoxazole MIC_50_ value of porcine isolates, while companion animal isolates exhibited 1 to 2 dilution steps higher MIC_50_ or MIC_90_ values of amoxicillin/clavulanic acid and cephalosporins. However, in consideration of the limited number of companion animal isolates available for the present study,it can only be assumed that divergences in MICs reflect differences in treatment of farm and companion animals. A different situation was seen for the aminoglycoside antibiotic streptomycin, which is generally not recommended for treatment of respiratory infections in swine. However, MIC_90_ values of porcine isolates were at least 3 dilutions steps higher than MICs of isolates obtained from companion animals. As previously reported for *Pasteurella* and *Mannheimia* isolates, streptomycin resistance genes are frequently linked to sulfonamide resistance genes, offering the possibility of co-selection [57]. Since almost all *strA*-carrying *B*. *bronchiseptica* isolates were also postive for *sul2*, co-selection imposed by the use of sulfonamides might explain the frequent occurrence of *strA* resistance genes in our strain collection.

Reading the MIC values of porcine *B*. *bronchiseptica* after 24 hours of incubation has recently been shown to be advantageous over reading the values after 16 to 20 hours incubation due to a higher reproducibility of broth microdilution susceptibility testing results [30]. This finding from *B*. *bronchiseptica* was in good accordance with many fastidious or special problem veterinary pathogens, for which modified incubation times are recommended in CLSI standards, including *Actinobacillus pleuropneumoniae* and *Histophilus somni* [29]. In a recently published study of *B*. *bronchiseptica* isolates from humans, MIC_50_ and MIC_90_ values of isolates were compared after 24 and 48 hours incubation and an increase in MICs of one or two dilutions steps for most antimicrobial agents was detected when using the 48 hours incubation [14]. Apparently, the authors supposed an advantage in a longer incubation time for *B*. *bronchiseptica* isolated from humans as well. Thus, this study was aiming to compare the MIC_50_ and MIC_90_ key figures and the classification of isolates into the categories susceptible and resistance after 20 hours and 24 hours incubation. By investigating a larger number of *B*. *bronchiseptica* isolates from swine and companion animals, it became apparent that reading the results after 24 hours incubation would not lead to a different classification of all 107 isolates from swine for ampicillin. For florfenicol, there was no change in classification after 24 hours incubation from susceptible or intermediate to resistant for all 107 isolates of porcine origin. A shift from the category florfenicol susceptible to intermediate was detected in only ten porcine isolates, indicating that CLSI approved breakpoints are applicable even with use of a 24 hours incubation time. A further comparison of the categorization of isolates was precluded due to the lack of *B*. *bronchiseptica* breakpoints for most antimicrobials. As mentioned previously, the MIC_50_ and MIC_90_ values were higher for some antimicrobial agents after 24 hours of incubation, however, investigation of a larger number of isolates, as shown here, revealed that differences did not exceed one dilution step [30].

Analysis of the antibiotic resistance genes present in this strain collection showed that 146 isolates with an ampicillin MIC of ≥2 μg/ml carried the species-specific *bla*_BOR-1_ β-lactamase gene, hinting at a frequent occurrence of this chromosomally located gene in *B*. *bronchiseptica*. As previously reported [26], the presence of a second β-lactamase gene seems to increase MIC values of ampicillin. However, a single isolate with low MIC of ampicillin was also positive for *bla*_OXA-2_, suggesting that an incomplete or functionally inactive gene may be present in this isolate. Although a single porcine isolate was classified as florfenicol resistant and three companion animal isolates showed elevated MICs, none of the tested phenicol resistance genes were detected, suggesting that a so far unknown resistance mechanism mediates decreased resistance to florfenicol in these isolates. Resistance genes *strA* and *strB* are frequently linked in gram-negative bacteria and are often found on broad-host-range nonconjugative plasmids [58–60]. In this study, both resistance genes were found together on plasmids varying in size and, hence, providing the option of a further dissemination in *B*. *bronchiseptica*. A situation similar to the *strA* and *strB* genes has been reported for the *tet*(A) tetracycline resistance gene, the only class detected in our strain collection. Class A tetracycline resistance genes were found in many gram-negative bacteria such as *E*. *coli*, *Aeromonas hydrophila*, *Salmonella enterica* and *B*. *bronchiseptica*, presumably due to the involvement of Tn*1721* and Tn*1721*-like elements in their dissemination and a frequent association with conjugative plasmids [61–63]. Interestingly, genes mediating resistance to sulphonamides (*sul1* and *sul2*) were widely-distributed in our strain collection. However, the occurrence of the genes was not clearly associated with elevated MICs of the folate inhibitor combination trimethoprim/sulfamethoxazole and, unfortunately, a sulphonamide antibiotic alone was not included in the tested panel of antimicrobial agents. Elevated MICs were detected only in isolates carrying both, the *sul1* and *dfrA7/A17* resistance genes. These findings may underline the synergistic activity of both antimicrobial agents, even though the functionality of the *sul1* and *sul2* genes still need to be demonstrated [64].

## Conclusion

The study demonstrated that current *B*. *bronchiseptica* isolates tested in this study are susceptible to a broad range of veterinary relevant antimicrobial agents. For penicillins and cephalosporines, higher resistance rates or intrinsic resistances were present. Modifying the incubation time from 16–20 hours to 24 hours as recently proposed for broth microdilution susceptibility testing of *B*. *bronchiseptica* to obtain the most consistent MIC values would lead to slightly higher MIC values with a maximum of one dilution step. No change in classification of isolates from florfenicol susceptible or intermediate to resistant was detected in 107 porcine isolates, indicating an applicability of CLSI approved breakpoints for *B*. *bronchiseptica* even with use of a 24 hours incubation time.
